# Modification of Physio-Mechanical Properties of Chitosan-Based Films via Physical Treatment Approach

**DOI:** 10.3390/polym14235216

**Published:** 2022-11-30

**Authors:** Endarto Yudo Wardhono, Mekro Permana Pinem, Sidik Susilo, Bintang Junita Siom, Agung Sudrajad, Agus Pramono, Yenny Meliana, Erwann Guénin

**Affiliations:** 1 Faculty of Chemical Engineering, University of Sultan Ageng Tirtayasa, Jl. Jendral Sudirman km 3, Cilegon 42435, Banten, Indonesia; 2 Laboratorium Polimer dan Komposit, Centre of Excellent, University of Sultan Ageng Tirtayasa, Jl. Jendral Sudirman km 3, Cilegon 42435, Banten, Indonesia; 3 Faculty of Mechanical Engineering, University of Sultan Ageng Tirtayasa, Jl. Jendral Sudirman km 3, Cilegon 42435, Banten, Indonesia; 4 Faculty of Metallurgical Engineering, University of Sultan Ageng Tirtayasa, Jl. Jendral Sudirman km 3, Cilegon 42435, Banten, Indonesia; 5 Research Center for Chemistry, National Research and Innovation Agency, BRIN, Kawasan Puspiptek, Serpong, South Tangerang 15314, Banten, Indonesia; 6 Integrated Transformations of Renewable Matter Laboratory (EA TIMR 4297 UTC-ESCOM), Université de Technologie de Compiègne, rue du Dr Schweitzer, 60200 Compiègne, France

**Keywords:** chitosan, polyvinyl alcohol, blend films, film casting, physical cross-linking

## Abstract

The premise of this work is the modification of the properties of chitosan-based film for possible use in food packaging applications. The biofilm was prepared via thermal and mechanical treatment through blending polymers with chitosan using Polyvinyl Alcohol (PVA) and loading different types of chemical agents, i.e., citric acid (CA), succinic acid (SA), and tetraethoxysilane (TEOS). The modification was carried out under high-speed homogenization at elevated temperature to induce physical cross-linkage of chitosan polymer chains without a catalyst. The findings showed that PVA improved the chitosan films’ Tensile strength (TS) and elongation at break (Eb). The presence of chemicals caused an increase in the film strength for all samples prepared, in which a 5% *w*/*w* of chemical in the optimum composition CS/PVA (75/25) provided the maximum strength, namely, 33.9 MPa, 44.0 MPa, and 41.9 MPa, for CA-5, SA-5, and TEOS-5, respectively. The chemical agents also increased the water contact angles for all tested films, indicating that they promoted hydrophobicity. The chemical structure analysis showed that, by incorporating three types of chemical agents into the CS/PVA blend films, no additional spectral bands were found, indicating that no covalent bonds were formed. The thermal properties showed enhancement in melting peak and degradation temperature of the blend films, compared to those without chemical agents at the optimum composition. The X-ray diffraction patterns exhibited that PVA led to an increasing crystallization tendency in the blend films. The morphological observation proved that no irregularities were detected in CS/PVA blend films, representing high compatibility with both polymers.

## 1. Introduction

Biofilms are thin layers made of bio-based polymers derived from renewable resources. The film acts as a functional barrier between food and the external environment to prevent moisture loss and suppress gas transfer, which encourages extended shelf life, improved quality, and reduced waste [1,2]. Chitosan is a versatile material with wide industrial applications [3,4], generally derived from the deacetylation of the chitin of marine crustaceans [5]. Chitosan provides some advantages, such as low toxicity, high biocompatibility, proliferation, and non-antigenicity, so it has potential for use as a preservative film, because it has good film-forming ability [6]. Unfortunately, brittleness in the material and sensitivity to moisture restrict its application in food packaging [7]. Blending synthetic and natural polymers is one strategy to produce a new class of materials since a single material may not have all the desirable flexibility for packaging film. Polyvinyl alcohol, PVA, is a synthetic water-soluble polymer with wide industrial applications and can be used as a blending material for biofilm composite preparation with several natural polymers due to its unique properties [8,9]. PVA is a semi-crystalline, harmless material with good mechanical properties and high biocompatibility [10], having numerous hydroxyl functional groups in molecular chains [11]. PVA is potentially miscible with chitosan since chitosan has abundant amine and hydroxyl groups, facilitating a homogeneous blend of polymers by forming hydrogen bonds [9]. The goal of blending chitosan with PVA from a functionality standpoint is to improve, customize, and maximize chitosan-based film performance. Many studies were performed on improving properties by blending chitosan with PVA, describing their diverse applications [12,13]. Kadir et al. used plasticized chitosan/PVA blend with ethylene carbonate, doped with ammonium nitrate, as an electrolyte in a battery, enhancing the conductivity of the system [14]. In another work, adding formaldehyde and glycerol as cross-linking agents and plasticizers improved the thermal stability and strength of the chitosan/PVA blend [15]. Tripathi used glutaraldehyde as across-linker agent in developing chitosan/PVA blend film as microbiological screening against food pathogenic bacteria, using the solution casting method [16].

Polymer blends involve modification processes to produce a new material with different properties by simple mechanical blending of different polymers [17]. This is an effective technique to address various formulations by combining favorable properties of different polymers, based on their end-use application, and is economically advantageous over synthesizing new materials. Polymer blends are categorized into heterogeneous and homogeneous blends. Heterogeneous blends are immiscible in multi-phase mixtures, while the homogeneous ones are miscible in single-phase [18]. Phase separation is considered the main factor affecting the result of formulation, which is influenced by the characteristics of the polymers and the ratio, the type and the condition of the blending process, solvent, and other ingredients in the formulation.

To get an integrated network, the cross-linking method can be used to improve the properties of the biopolymer, particularly those originating from carbohydrates or proteins, by either physical or chemical interaction in the polymer chains [19,20]. The method involves the formation of covalent bonds between different chains, attaching or cleaving chemical groups, which reduces their mobility, so as to alter the solubility or other properties of the original molecule. Chemical cross-linking can overcome inherent deficiencies by forming intermolecular interactions, which improve mechanical and barrier properties [21]. Some materials were reportedly used as cross-linked agents of bio-based polymers, including glutaraldehyde [22], genipin [23], and phosphoryl chloride [24]. Physical cross-linking is obtained via mechanical treatment to improve intermolecular interactions. When the polymer solution is heated or shear stressed, it disrupts the hydrogen bonding among polymer chains. More open structures are produced, allowing more chain-to-chain interactions [25].

In this work, the characteristics of chitosan-based film were modified via physical treatment by blending with PVA. The idea was to avoid the use of chemical cross-linkers, that are either expensive or poisonous and do not lead to the desired properties of the final film. The modification was carried out under high-speed homogenization at elevated temperature to induce physical cross-linkage of chitosan polymer chains by introducing different types of chemical agents, i.e., citric acid (CA), succinic acid (SA), and tetraethoxysilane (TEOS), without a catalyst, for possible use in food packaging applications. This series process was expected to modify the properties of the chitosan/PVA blending solution through simultaneous turbulence, shear heating, and cavitation. The film formation was prepared using solvent evaporation and the casting procedure. In addition, glycerol was added as a plasticizer to improve the flexibility. The effects of various chemical agents on the film’s strength and water resistance are discussed.

## 2. Materials and Methods

### 2.1. Materials

Shrimp shell chitosan (average molecular weight, MW: 50–100 kDa; degree deacetylation, DD: 75–85%) was purchased from Sigma Aldrich, Singapore, Singapore. PVA (MW: 145,000 was obtained from Sigma Aldrich, Singapore. Chemical agents (CA, SA, and TEOS) were purchased from a local supplier, Maxlab, Tangerang, Indonesia. Glycerol (purity > 85%) was from Merck, Darmstadt, Germany. Acetic acid glacial was supplied by Rofa Chemical, Jakarta, Indonesia. Aquadest was produced by means of the demineralized water system at the polymer and composites laboratory, Universitas Sultan Ageng Tirtayasa, Cilegon, Banten, Indonesia. All the materials used were laboratory-grade without further purification.

### 2.2. Methods

Film casting methods prepared Chitosan/PVA polymer blends with different weight ratios. Step by step, the film preparation and characterization were as follows.

#### 2.2.1. Blending Solution

Polymer blends were prepared by mixing a solution of chitosan and PVA with different weight ratios under high-speed homogenization. Chitosan solution (CS) was produced by dissolving 1% *w*/*v* of chitosan powder in 0.15 M acetic acid using magnetic stirring at 1.000 rpm for 120 min at room temperature. The obtained CS was filtered using filter paper to remove the impurities. The PVA solution was prepared by dissolving 1% *w*/*v* of PVA in hot water at 80 °C using a mechanical stirrer at 3.000 rpm until a clear solution was obtained, and then it was cooled at room temperature before formulation. PVA and glycerol were consecutively introduced drop-wise and homogenized at 10.000 rpm for 15 min using a high-speed rotor-stator (D-160, BIOBASE, Jinan, Shandong, China). The blending solutions contained glycerol 10% *w*/*w* by a corresponding weight of dried CS/PVA films and PVA on different weight ratios (0–100 *w*/*w* on a dry basis of chitosan) to evaluate the optimum ratio through the strength.

#### 2.2.2. Physical Cross-Linking

A specified quantity of chemical reagents (0–7.5% *w*/*w* on a dry basis of dried CS/PVA films) was added to the optimum ratio of the blending solution and heated to 80 °C for 15 min. The mass and molar ratio of chemical reagents in the dried blend film is presented in Table 1.

#### 2.2.3. Film Casting Procedure

The film forming solution was poured into 9 × 9 cm^2^ plastic Petri dishes to cast into films. The solution was held in an air circulating chamber with Relative Humidity, RH: 60% and temperature (25 °C) for 48 h before peeling off the films. The transparent film samples were stored in a drying chamber at 50% RH for further characterization.

### 2.3. Film Characterization

- Tensile strength (TS) and elongation at break (Eb) of the films were measured using a universal testing machine (Strograph, Toyo Seiki, Tokyo, Japan) with a preload cell of 0.1 MPa and a speed tensile modulus of 50 mm/min. The grip to grip separation at the start position was set at 50 mm. Rectangular specimens (8 cm × 1 cm × 0.05 cm) were produced with three samples, each measuring cut from the cast films. The TS (MPa) was calculated by dividing the maximum load on the film before failure by the cross-sectional area (m^2^) of the initial specimen. The samples were conditioned for at least 48 h in a drying chamber (25 °C and 50% RH).

The measurements were repeated three times for the TS and Eb. The data presented here fell under the 95% confidence interval.

- Surface wettability of the film was evaluated by the contact angle (CA) using a Drop Shape Analyzer (DSA 100; Kruss GmbH, Hamburg, Germany). The measurement was carried out through the static droplet method, in which one drop of water fell on the film’s surface. A video camera recorded the droplet’s shape to determine the contact angle.

The measurements were repeated five times for the contact angle measurement. The data presented here fell under the 95% confidence interval.

- The chemical structure of the films was characterized using Fourier-transform infrared spectroscopy (FTIR), with a Nicolet iS5 spectrometer (Thermo, Waltham, MA, USA) at ambient temperature. Data were collected over 32 scans at a 4 cm^−1^ resolution, ranging from 400 to 4000 cm^−1^.
- Thermal properties were analyzed by Differential scanning calorimetry (DSC), (Q100, TA Instruments, New Castle, DE, USA). The sample film was placed in an aluminum crucible with less than 20 mg. DSC curves were obtained by heating the samples from 0 to 300 °C, with a heating rate of 10 °C/min, under a nitrogen gas flow of about 50 mL/min.
- The diffraction pattern of the sample films was recorded using X-Ray Diffraction (XRD), in a D8 Advance (Bruker, Billerica, MA, USA). The films were scanned at a scanning angle of 2θ from 5 to 45° with CuKa filter radiation, where θ was the incident angle of the X-ray beam on the sample.
- The cross-sectional image of the films was observed by scanning electron microscopy (SEM), (JEOL-2100F, JEOL Ltd., Tokyo, Japan). The films were frozen in liquid nitrogen and were cut later. The cross sections were sputter-coated with a gold layer about 10 nm thick to avoid charging under the electron beam.

## 3. Results

### 3.1. Mechanical Properties

**Figure 1:**
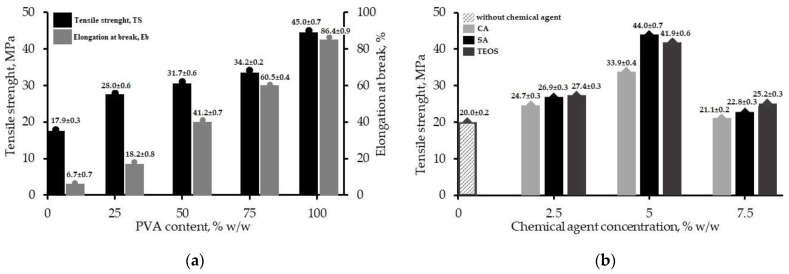
The effect of the blend film composition on the maximum strength of CS/PVA blend with varying weight ratio (**a**); Optimum composition film blend with varying mode of chemical agents (**b**).

The blend films of CS and PVA were prepared in various proportions to find the optimum ratio of the film’s mechanical properties, i.e., strength and elongation, containing the minimum composition of the synthetic material (PVA) in the blend films. The CS/PVA blends were composited at different weight ratios of PVA from 0 to 100% *w*/*w*. Figure 1a shows the film test reports, which were determined according to TS and Eb.

The mechanical properties of the CS film were measured at 17.9 ± 0.3 MPa and 6.7 ± 0.7%, while the film of only PVA was measured at 45.0 ± 0.7 MPa and 86.4 ± 0.9%, respectively. The presence of PVA improved the CS films’ TS and Eb, in which the strength and toughness of the investigated blend films tended to increase as the PVA content increased [15,26,27,28]. The blends at different ratios showed similar trends. The film containing 25% PVA recorded TS as 28.0 ± 0.6 MPa. Further, it was gradually augmented at 31.7 ± 0.6 MPa (50%) and 34.2 ± 0.2 MPa (75%). Meanwhile, regarding elongation, the yield at the break value ranged from 18.2 ± 0.8% (25%) to 41.2 ± 0.7% (50%) and 60.5 ± 0.4% (75%) as the amount of PVA increased. Concerning the CS/PVA blend films that were treated by a different mode of chemical agents, CA, SA, and TEOS, the values of the strength are presented in Figure 1b. The optimum film composition was labeled according to the chemical concentrations, for example, CA-5, SA-5, and TEOS-5. The chemical agents enhanced the TS of the films for all types used. The results of TS at 2.5% *w*/*w* were 24.7 ± 0.3 MPa (CA-2.5), 26.9 ± 0.3 MPa (SA-2.5), and 27.4 ± 0.3 MPa (TEOS-2.5). Concentration of the chemicals at 5% *w*/*w* provided the maximum strength for all samples, namely, 33.9 ± 0.4 MPa; 44.0 ± 0.7 MPa; and 41.9 ± 0.6 MPa, for CA-5, SA-5, and TEOS-5, respectively. Moreover, the concentrations above 5% decreased the TS of the films significantly, i.e., 21.1 ± 0.2 MPa with CA-7.5, 22.8 ± 0.3 MPa with SA-7.5, and 25.2 ± 0.3 MPa with TEOS-7.5.

### 3.2. Water Contact Angle

**Figure 2:**
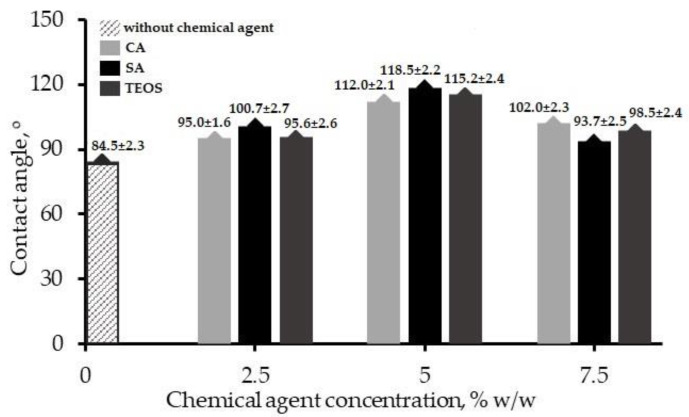
The effect of physical treatment on the contact angle of the film surface.

Water contact angle (WCA) was measured to assess the surface wettability of the films. It was a simple technique to evaluate whether the blend films have a hydrophobic or hydrophilic character. Experimentally measured contact angles between the CS/PVA film surface and the dropping water are shown in Figure 2.

As presented in the Figure, the WCA of the film (0% *w*/*w*) was 84.5 ± 2.3°. The addition of 2.5% chemicals increased the contact angles for all tested films, namely 95.0 ± 1.6° (CA-2.5), 100.7 ± 2.7° (SA-2.5), and 95.6 ± 2.6° (TEOS-2.5), indicating that the reagents used in this work promoted hydrophobicity. The WCA reached relatively high values ranging from 110 to 120° at 5% *w*/*w*: 112.0 ± 2.1°, 118.5 ± 2.2°, and 115.5 ± 2.4° for CA-5, SA-5, and TEOS-5, respectively. The angles further decreased by increasing the chemical reagents content at 7.5% *w*/*w*, where the WCA were down to 102 ± 2.3° (CA-7.5), 93.7 ± 2.5° (SA-7.5), and 98.5 ± 2.4° (TEOS-7.5).

### 3.3. FTIR Analysis

**Figure 3:**
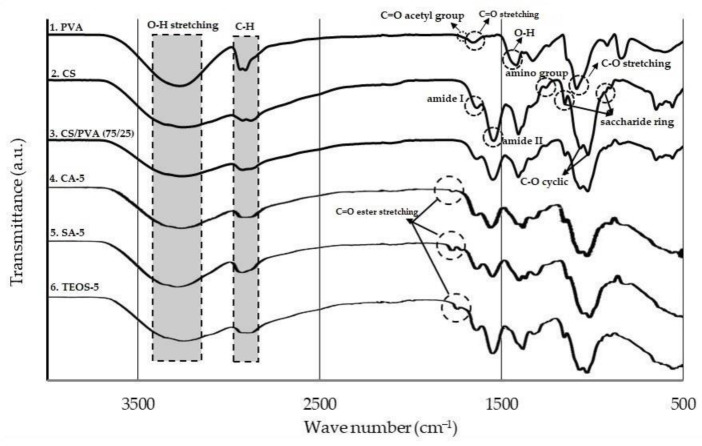
FTIR spectra of: (1) PVA; (2) CS; (3) blend film of CS/PVA (75/25); (4) blend film of CA-5; (5) blend film of SA-5; (6) TEOS-5.

FTIR spectroscopy was applied to characterize the presence of specific chemical groups and monitor the functional group alteration. It was a suitable technique for investigating molecular hydrogen-bonding interactions from the blend CS/PVA films. The spectra of the PVA, CS, and films are shown in Figure 3 and the summary of the vibration bands are listed in Table 2.

### 3.4. DSC Test

**Figure 4:**
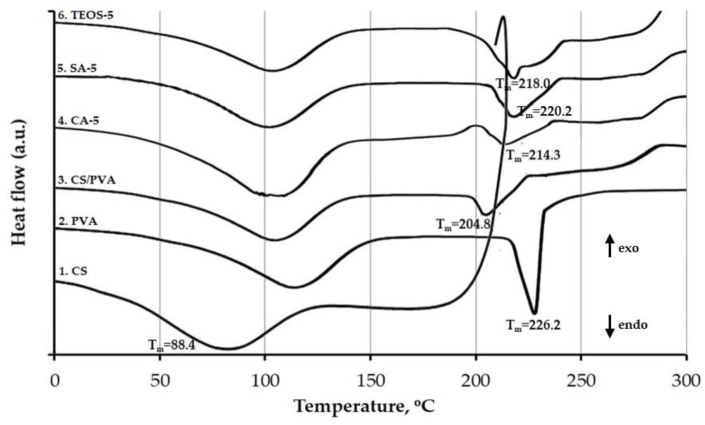
DSC thermograms of: (1) CS; (2) PVA; (3) blend film of CS/PVA (75/25); (4) blend film of CA-5; (5) blend film of SA-5; (6) TEOS-5.

The thermal characteristics of the films were analyzed by measuring melting temperature, Tm, to investigate the crystallinity of polymers and temperature degradation, Td. [29,30]. The DSC plot between heat flow and temperature for the samples is shown in Figure 4.

The DSC curves of all sample films appeared to have broad endothermic peaks in the temperature range from 50 °C to 150 °C. This was related to the moisture content associated with the sample films [31]. The signal of the CS showed the endothermic peak was distributed at 25–125 °C with the onset at 88.4 °C and a sharp exothermic peak starting at 210.0 °C, related to the structural decomposition of chitosan [32].The PVA displayed two successive endothermic peaks. The first was at around 116.7 °C, assigned to the dehydration during heating, and the other at a peak maximum of 226.2 °C, the melting transition [33]. The sample CS/PVA was observed to have a significantly smaller melting peak than the pure PVA. The onset of the melting peak shifted to a lower temperature at 204.2 °C from 226.2 °C. The melting depression might be related to the rigidity of the polymer, which was associated with morphological effects, such as crystallinity index [34]. Decrease of the melting peak due to the presence of chitosan in the blending system indicated a reduction of crystalline phase and an enlargement fraction of amorphous phase [30]. The reduction of the crystallinity or melting temperature indicated the blend film’s miscibility [8,35].

### 3.5. XRD Measurement

**Figure 5:**
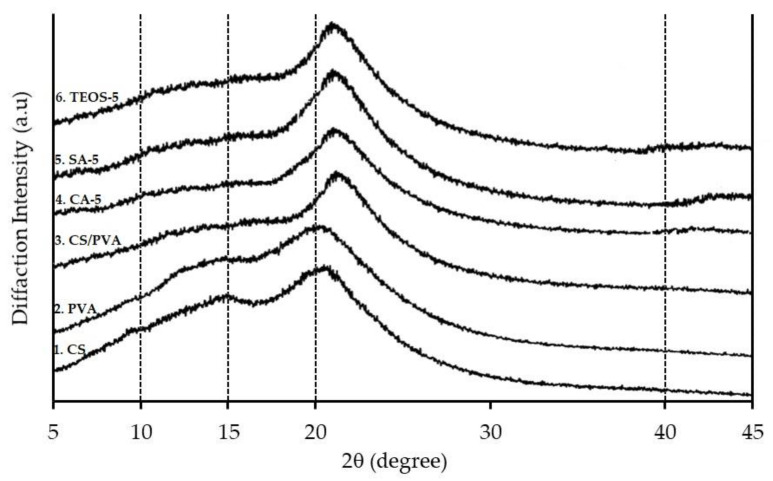
XRD pattern of: (1) CS; (2) PVA; (3) blend film of CS/PVA (75/25); (4) blend film of CA-5; (5) blend film of SA-5; (6) TEOS-5.

Figure 5 presents the diffraction patterns of CS, PVA, CS/PVA blend film, and CS/PVA with different loadings of chemical agents, i.e., CA-5, SA-5, and TEOS-5 under study.

The CS film exhibited two prominent peaks around 15.0° and 20.4°, indicating a primarily amorphous structure in chitosan [36]. The crystallinity index (CrI) was calculated by a simple ratio of the crystalline area associated with the reflections (200) and (314) [37] to the total area ranging from 5° to 45° [38], which was 18.1%. At the same time, the PVA presented a similar diffraction pattern with two broad peaks at 11.8° and 20.0°. The diffraction pattern indicated that the PVA had a semi-crystalline structure. The average crystallinity of PVA film was 22.8%. These results were in accordance to the literature [39,40].

### 3.6. SEM Observation

**Figure 6:**
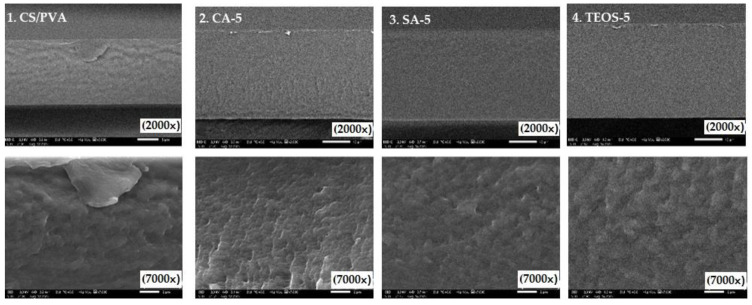
SEM images of blend films: (**1**) blend film of CS/PVA (75/25); (**2**) blend film of CA-5; (**3**) blend film of SA-5; (**4**) TEOS-5.

The microstructure of the blend films was qualitatively observed through SEM. The micrographs of the film cross sections of all formulations are shown in Figure 6.

## 4. Discussions

### 4.1. Quantitative Analysis

From the mechanical test point of view, it seemed PVA improved the blend strength in any composition, which could be due to the interaction between (–OH) and (–NH_2_) groups of CS and –OH groups of PVA [41] restricting the motion of the matrix while promoting rigidity [42]. Several works reported the same trends, where chitosan-based blend films were blended with PVA to improve its properties. Olewnik-Kruszkowska et al. prepared PVA and CS antibacterial films with the addition of polyhexamethylene guanidine (PHMG). The findings showed that the strength of CS film and CS/PVA blend films was lower than pure PVA [26]. Abraham et al. blended chitosan with PVA to enhance the thermal stability of the blend films. The results proved that the strength and elasticity of the CS/PVA blend films reduced with a decrease in PVA content [15]. Furthermore, Nugraheni et al. evaluated the physical properties of CS/PVA films with calcium chloride. They found that domination of PVA concentration increased the strain and tensile stress of the CS/PVA blend films [28]. Accordingly, the ratio of CS/PVA (75/25) film was chosen for the subsequent experiments.

**Figure 7:**
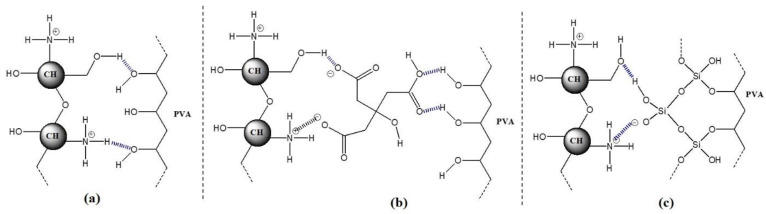
Schematic representation of the formation of intermolecular hydrogen bonding between CS and PVA with chemical agents: citric acid (**a**); succinic acid (**b**); TEOS (**c**).

The results of the mechanical test indicated that the chemical additives induced interfacial interaction with the matrix CS/PVA, owing to the formation of hydrogen bonds [43,44]. In this case, the non-covalent interaction of molecules is the primary approach to be considered to increase the mechanical properties of CS/PVA blends. According to Nicolle et al. [45], chitosan-based materials can be modified in their characteristics via non-covalent attachment by combining with small molecules, proteins, and polymers. Under this technique, the characteristics of chitosan depend on non-covalent interactions, such as hydrogen bonds, ionic bonds, and chelation. The addition of small negatively charged molecules into the host matrix triggers a gelation process, in which the additive molecules and the matrix chains arrange themselves into an amorphous hydrogel [46]. Interestingly, the mechanical strength of the chitosan can be improved by its non-covalent association with specific chemical precursors [47,48]. The non-covalent interaction is built due to the formation of hydrogen bonds between CS and polymer PVA with chemical agents, and is presented in the schematic representation in Figure 7.

Based on the data in Figure 1b, there was not enough non-covalent interaction between the polymer molecules to improve the TS at the lower chemical additive concentrations. Meanwhile, at the higher ones, excess chemical limited the mobility of the molecules, leading to a decrease in strength [49,50]. The chemical also caused an increase in the film strength, which could modify the polymer network among the molecular chains, and provide better interconnection between molecules than the chemical ones [51]. In this case, SA appeared to display the best impact, compared to CA and TEOS. Furthermore, this simple approach for chitosan-based film modification showed the desired mechanical properties, which were as good as the ones developed with covalent interaction using chemical cross-linkers that are expensive [52,53] and toxic [54,55] for food packaging application. The comparison results to the literature are shown in Table 3.

From the WCA analysis, the results showed that the blend films exhibited reduced wettability. Surfaces with a contact angle over 90° were indicated as hydrophobic. In this case, the presence of additives changed the wetting behavior of the blend films. This was because of the close packed structure and the fact that free functionals were unavailable, due to small negatively charged additive molecules entrapped in the CS/PVA matrix induced by non-covalent bonding with the amino groups of CS chains. Very few functional groups were available for hydrogen bonding and increasing ionic cross-linking due to the increasing quantity of additives. Thus, the hydrophilic character shifted towards a hydrophobic character [57]. This accorded with the work of Khan et al. [58], where CS/PVA/graphene oxide hydrogel composites were produced using the solution casting method with TEOS as the cross-linker agent. The hydrogel composites presented improved WCA by increasing TEOS quantities, which changed the wettability of the sample from hydrophilicity to hydrophobicity. At the same time, Ali et al. [59] prepared a thin film based on the composites CS and polyvinylpyrrolidone (PVP) cross-linked with maleic acid for membrane desalination application. The WCA improved with increase in maleic acid concentration from 0.5% to 2.5%. A decrease in matrix CS/PVP pore size with an increase in the quantity of maleic acid, in which less water could go through the membrane, resulted in a higher WCA value. According to Heydari [60], forming physical cross-linked bonds which hold portions of several polymer chains together improves the film’s mechanical and water resistance properties. In contrast, excess chemicals seemed to form aggregates and reduced polymer mobility, resulting in a lower WCA. In this work, SA-5 produced the highest WCA compared to the others.

### 4.2. Qualitative Analysis

In FTIR analysis, there was, typically, a strong hydroxyl band in the region from 3450 to 3600 cm^−1^ for the non-bonded -OH and the band for hydrogen-bonded -OH was at 3200–3570 cm^−1^ [30]. For the PVA, the broadband at around 3450 cm^−1^ was characterized by O-H stretching vibration, and at the positions 1430 cm^−1^ corresponded to OH bending of the hydroxyl group [61]. The vibration band at 1108 cm^−1^ was due to C-O stretching [62]. The band corresponded to the C=O acetyl group found at 1758 cm^−1^, which presented as the PVA backbone, and the one related to C-H methylene group asymmetric stretching vibration occurred at 2930 cm^−1^ [63]. For the CS, the absorption bands at 890 and 1150 cm^−1^ corresponded to the saccharide structure, and the peak at 1250 cm^−1^ corresponded to the amino group of chitosan. The vibration bands at 1558, 1658 and 3430 cm^−1^ were assigned to Amide II, Amide I and O-H stretching bands, respectively [61]. For the CS/PVA blend films, the FTIR spectra appeared around 3380 cm^−1^, which was attributed to an O-H stretching of PVA with a secondary amine N-H of chitosan. This absorption indicated the interaction between PVA and CS through hydrogen-bonding [61]. In these spectra, the peak at 1250 cm^−1^ disappeared, and the band found at 1077 cm^−1^ indicated the presence of the O-H group with polymeric association and the secondary amine N-H [61]. For sample films containing chemicals: CA-5, SA-5, and TEOS-5, the small peaks that appeared at 1720 cm^−1^ might be attributed to the C=O ester stretching from PVA [64]. In this case, incorporating three types of chemical agents into the CS/PVA blend films, meant no additional spectral bands were found, indicating that no covalent bonds were formed. The shifting of absorption peaks in the blend films might result from the interaction of all compounds ascribed to physical response.

The DSC curves of the samples with 5% *w*/*w* of chemical agent showed no significant shift. The DSC results ratified the results accomplished by FTIR, in which the peak types in the thermograms resulted from physical response during the film preparation. However, the incorporation of chemicals presented an increase in melting peak compared to that without chemicals, namely ΔT = 10.1° for CA-5, ΔT = 16.0° for SA-5, and ΔT = 13.8° for TEOS-5. According to Mao et al., the strong interaction between molecular chains was stably formed by electrostatic association and hydrogen bonds to render breakage and enhance compatibility of the blend film [65]. The exothermic peak was found to start at 273.8 °C. The peak was ascribed to the thermal degradation of the films, which occurred at 281.1 °C (CA-5), 283.3 °C (SA-5), and 282.1 °C (TEOS-5), respectively, suggesting that the thermal stability was considerably improved. In this case, the physical treatments that caused the improvement in molecular chain interaction between chitosan and PVA were suggested to form more stable films.

In XRD measurement, the interaction between CS and PVA molecules was shown in CS/PVA blend film ratio (75/25). The peak of PVA at 11.7° disappeared, and the peak of the CS at 20.4° slightly shifted to 21.8°. The presence of PVA led to increasing crystallinity, in which the CrI of the blend films went up by 20.1%. In general, the crystalline structure was an independent variable which influenced important properties, such as mechanical strength and thermal properties [66]. This result concluded that strong interaction formed by hydrogen and ionic bonding occurred between chitosan and PVA molecules in the blend films at the composition prepared. Generally, a weak molecular interaction produced its diffraction peak for each material, expressed as heterogeneous mixed peaks in the blend films [35]. Furthermore, the diffractogram of the blend films reticulated with three types of chemicals (CA-5, SA-5, and TEOS-5) did not show any significant change in peak positions or in Crl, compared to the blend film without chemical reagent (CS/PVA).

From SEM observation, homogeneous blends could be observed in all samples prepared without phase separation at magnification of 2000×. The morphology of the film indicated that a close packed structure was obtained. No irregularities were detected in the blend films, such as air bubbles, pores, cracks or droplets, indicating high compatibility with both polymers. This was beneficial for the mechanical properties and water resistance because it indicated an improved phase adhesion. However, with a clear change in the samples with magnification 7000×, the structure of the CS/PVA blend (Figure 6.1) appeared to have fewer cracks at the cross-sectional. At the same time, CA-5, SA-5, and TEOS-5 films looked homogeneous, due to better interaction between both polymers in the presence of chemical agents.

## 5. Conclusions

The chitosan-based film was successfully modified by a simple approach with physio-mechanical properties via mechanical and thermal treatment to induce physically cross-linking. The modified chitosan showed desired mechanical properties, that were as good as the film developed with chemical cross-linking. The results showed that blending chitosan with PVA, associated with different chemical agents (citric acid, succinic acid, and tetraethoxysilane), improved the overall properties of CS/PVA blend films. The modified films’ characteristics displayed a significant enhancement of film strength at the optimum ratio of chitosan to PVA (75/25) with 5% *w*/*w* of chemicals, namely, 33.9 MPa (CA-5), 44.0 MPa (SA-5) and 41.9 MPa (TEOS-5). The chemical agents also increased the water contact angles, ranging from 84.5° (without chemicals) to 112.0°, 118.5°, and 115.5° for CA-5, SA-5, and TEOS-5, respectively, indicating that they promoted hydrophobicity. The chemical structure analysis by FTIR proved that the films had a new characteristic band that might be attributed to the non-covalency between chitosan and PVA. The combination of chitosan and PVA rendered films of homogeneous blend, due to the high compatibility of both polymers, as revealed by XRD, DSC, and SEM tests.

## Figures and Tables

**Table 1:** The ratio of chemical reagent in the dried CS/PVA blend films.

Chemical Reagent	Ratio %					
	Mass	Molar	Mass	Molar	Mass	Molar
CA	2.5	10.6	5.0	19.3	7.5	26.4
SA	2.5	16.2	5.0	28.0	7.5	36.8
TEOS	2.5	9.9	5.0	18.0	7.5	24.8

**Table 2:** FTIR spectral peak of the blend film components.

Component	Wave Number, cm^−1^	Assignment
PVA	1108	C-O stretching
	1430	O-H bending
	1758	C=O acetyl group
	2930	C-H methylene group
	3450	O-H stretching
CS	890 & 1150	Saccharide structure
	1250	Amino group
	1558	Amide II
	1658	Amide I
	3430	O-H stretching
CS/PVA	1075	O-H group of PVA
	3380	O-H group of Chitosan
CA-5; SA-5; TEOS-5	1720	C=O ester stretching

**Table 3:** Mechanical strength of CS/PVA blend films as affected by chemical cross-linkers.

Ratio CS/PVA (wt%)	Chemical Agent	Tensile Strength, MPa	
		Exp. Results	Reference
75/25	Citric acid	33.9	-
75/25	Succinic acid	44.0	-
75/25	TEOS	41.9	-
50/50	Citric acid		29.7 [56]
50/50	Poly(Hexamethylene Guanidine)		62.0 [26]
75/25	Calcium chloride		33.53 [28]
75/25	Glutaraldehyde		25.3 [54]
